# A scoping review protocol of evidence-based guidance published by general practitioner professional organisations

**DOI:** 10.12688/hrbopenres.13268.1

**Published:** 2021-05-18

**Authors:** Emer O'Brien, Barbara Clyne, Susan M. Smith, Noirin O'Herlihy, Velma Harkins, Emma Wallace

**Affiliations:** 1 Department of General Practice, Royal College of Surgeons in Ireland, Dublin, D02H903, Ireland; 2 Irish College of General Practitioners, 4/5 Lincoln Place, Dublin 2, D02XR68, Ireland

**Keywords:** General practice, family practice, general practitioner, family practitioner, primary healthcare, practice guidelines, evidence based practice.

## Abstract

**Introduction: **General practitioners (GPs) strive to use a patient centred approach to achieve shared decision making by integrating clinical evidence, clinical judgement, and patient priorities. This protocol outlines a scoping review to identify what evidence-based guidance is produced by general practitioner professional organisations internationally to support general practice clinical decision making.

**Methods: **This scoping review will be conducted using the framework proposed by the Joanna Briggs Institute and the Preferred Reporting Items for Systematic Reviews and Meta-analysis extension for scoping reviews (PRISMA-ScR), will be used to guide the reporting. Two researchers will search electronic databases (Medline, Embase, Cochrane Library and Scopus), grey literature sources and contact international GP professional organisations directly to identify appropriate studies for inclusion. Key information will be categorised and classified to generate a summary of the methods used internationally to develop and implement evidence-based guides for general practitioners and a narrative synthesis will be conducted.

**Conclusions: **This scoping review will identify the role of GP professional organisations in generating, endorsing and/or disseminating evidence-based guidance for supporting general practitioner’s clinical decision making to benefit patient care.

## Introduction

General practitioners (GPs) require evidence-based guidance to support patient care ^1– 5^. GPs have a unique role in society, practicing medicine in the context of the family and community. Through the comprehensive, coordinated and continuous provision of care, GPs manage the physical, mental, emotional and social needs of their patients ^6^. GPs strive to use a patient centred approach to achieve shared decision making by integrating clinical evidence, clinical judgement, and patient priorities ^7, 8^.

Internationally, GP professional organisations produce evidence-based guidance for use by GPs in clinical practice. There are differences across countries as to how this evidence is developed and implemented. In the Netherlands, for example, the Dutch College of General Practice (NHG) develop *de novo* clinical guidelines for GPs ^9^. In comparison, GP professional organisations in other countries disseminate materials to GPs based on national or international guidelines as part of an adopt or adapt approach ^10– 12^. Factors influencing the role of professional GP organisations in developing and/or disseminating clinical guidelines include the role of central national guideline development agencies and the availability of resources ^13^. It may not be feasible for GP professional organisations to develop *de novo* clinical guidelines, which require evidence synthesis and the generation of recommendations ^14^.

The role of the GP in the healthcare system also has an impact. GPs in some countries have a specific gatekeeper role, authorising access to specialty care, hospital care, and some diagnostic tests. This gatekeeping role has crucial influences on optimising health service utilisation, health outcomes, healthcare costs, and patient satisfaction ^15^. GPs who are gatekeepers are more likely to require GP orientated guidelines.

Despite differing healthcare systems and roles of the GP, a challenge for many GPs is staying abreast of a wide breadth of changing evidence. With the growth of evidenced based medicine there has been a shift from opinion-based care delivery to a process that incorporates the best available evidence with clinical judgement or expertise, incorporating patient priorities ^16^. In a review of Australian clinical guidelines, there were nine times more clinical guidelines published in 2010 compared to 1993 ^17^. The challenge of staying up to date with the rapidly evolving evidence and increasing complexity of clinical care means that GPs need access to relevant and robust evidence sources as part of their continuing professional development.

Understanding the varied role of international GP professional organisations in generating and disseminating evidence-based guidance will help inform how GP professional organisations best support clinical decision making in the ‘unique ecology of general practice’ ^18^. The aim of this scoping review is to identify what evidence-based guidance is published by GP professional organisations internationally to support GPs in their clinical decision making with patients. The objectives are i) to identify the topics of these guidance documents, both clinical and non-clinical; ii) to review the methods used to develop evidence-based guidance and how these guidance documents are structured and, iii) to explore how evidence is disseminated to GPs.

## Methods

### Scoping review framework

This scoping review will follow the framework proposed by the Joanna Briggs Institute (JBI) ^19– 22^. While, the overall conduct of the scoping review is informed by the JBI framework, the Preferred Reporting Items for Systematic Reviews and Meta-Analysis extension for scoping reviews (PRISMA-ScR) will be used to guide the reporting of the scoping review ^23^.

As this is a scoping review it will be designed to identify the range of the evidence available and will be represented as a mapping of the identified data, without the act of synthesis or particular reference to methodological quality of relevant studies ^24^.

### Stage 1: Identifying the research question

Overarching question: What evidence-based guidance is published by general practice professional organisations to support GPs clinical decision making?

Objectives:

a. What are the content topics that the organisations are providing?

b. What are the methods for developing these guides?

c. What are the structures of the guides and how are they presented?

d. How are these guides disseminated to GPs?

### Stage 2: Identifying the relevant studies

Table 1 contains the eligibility criteria for the scoping review. Articles will be included where they are an evidence-based guidance document or guideline produced by a national general practice professional organisation. These guidance documents must support GPs clinical decision making and patient clinical care and be published in the last 10 years for currency. No language restrictions will be applied.

**Table 1.** Eligibility criteria for inclusion in the review.

Inclusion Criteria	Rationale
• Evidence based guidance developed following a comprehensive review of the literature	Any evidence-based guidance or guideline produced by general practitioner (GP) professional organisations to support GP clinical decision-making. In order to be considered evidence-based, guidance documents will be included where they explicitly state they are based on a review of the literature (including systematic reviews, scoping reviews, rapid reviews, narrative reviews) Must be peer reviewed, reviewed by committee or experts. Definition of Evidence based guidelines *‘systematically developed statements to assist practitioner decisions about appropriate* *healthcare for specific clinical circumstances’* Evidence based as they *‘use the results of systematic literature reviews in formulating the* *recommendations’* ^25^
• Published by General Practice professional organisations	Publications must directly target GPs
• National	Regional programmes excluded
• Publications within the last 10 years	Most relevant
• No language restriction	
• For the purposes of this review the following definition of general practice is used	‘ *General practitioners/family doctors are specialist physicians trained in the principles of the discipline. They are personal doctors,* *primarily responsible for the provision of comprehensive and continuing care to every individual seeking medical care irrespective* *of age, sex and illness. They care for individuals in the context of their family, their community, and their culture, always respecting* *the autonomy of their patients. They recognise they will also have a professional responsibility to their community. In negotiating* *management plans with their patients they integrate physical, psychological, social, cultural and existential factors, utilising the* *knowledge and trust engendered by repeated contacts. General practitioners/family physicians exercise their professional role by* *promoting health, preventing disease and providing cure, care, or palliation. This is done either directly or through the services of* *others according to health needs and the resources available within the community they serve, assisting patients where necessary in* *accessing these services. They must take the responsibility for developing and maintaining their skills, personal balance and values as a* *basis for effective and safe patient care’* ^26^.
• Exclude publications with </= 2 authors	
• Patient clinical care	Exclude guidance relating to practice management and other non- clinical topics

## Search strategy

The search strategy will identify both published and grey literature and will follow a three step strategy, as per JBI ^19^. A copy of the search strategy is shown in Table 2.

**Table 2.** Search strategy.

	Ovid MEDLINE(R) and Epub Ahead of Print, In-Process, In-Data-Review & Other Non-Indexed Citations, Daily and Versions(R) 1946 to March 09, 2021	
1	general practice.mp. or exp Family Practice/ or exp General Practice/	97226
2	(general ADJ1 practitioner) OR (general ADJ1 practitioners) OR (family ADJ1 practi?e) OR (family ADJ1 physician*)	126886
3	primary health care.mp. or exp Primary Health Care/	310657
4	1 OR 2 OR 3	
5	exp Practice Guideline/ OR exp Practice Guidelines as Topic/ OR (practice ADJ2 guideline$)	166143
6	((quick adj2 reference adj2 guide*) or (quick adj2 reference) or (evidence adj1 reference) or (evidence adj1 guide*) or ("evidence based" adj1 reference) or ("evidence based" adj1 guide)).mp.	1520
7	5 OR 6	167271
8	4 AND 7	12186
8	LIMIT 8 to 2010–2021	6175

The **first step**, the limited search, will include searching two appropriate online databases ( Medline and Embase). An analysis of the text words in the titles and abstracts of retrieved papers will be conducted, and of the index terms used to describe the articles.

The **second step** will use all identified key words and index terms to perform a second search of all the following databases: Medline, Embase, Cochrane Library and Scopus to identify peer reviewed research papers relating to our aim. This step will be conducted with input from an information specialist.

**Thirdly,** reference lists of included articles will be searched for additional relevant articles.

We foresee specific limitations to the above search. Word searching of databases may be inherently problematic as guides may not be reported in peer reviewed publications only. Therefore, they may not be retrieved or matched by only using this search strategy. Also, there is considerable heterogeneity of the nomenclature associated with this search, for example, guides versus guidance versus clinical guidelines. We plan to overcome these limitations by supplementing the search with targeted GP professional organisation contacts. This will be completed by contacting key informants in GP professional organisations, identified on the basis of the definition of general practice being used for the review and the role of the GP as a gatekeeper.

Grey literature search will also include searching ‘Guideline Central’ and ‘Evidence Search’.

The final included studies for screening will be downloaded to a reference management software package ( EndNote X9) and duplicates removed.

### Stage 3: Study selection

Titles and abstracts will be screened for inclusion against the inclusion criteria for the review ( Table 1). For those that appear to meet the inclusion criteria, full text articles will be retrieved and screened against the inclusion criteria. Those articles that fulfil all the inclusion criteria will be included in the review.

The above steps will be completed by two reviewers (EOB and SD). They will work independently initially and then come together to compare results. Any discrepancies will be resolved by consensus and if consensus is not reached will be referred to a third reviewer (EW).

Studies that do not meet the inclusion criteria will be excluded. Reasons for the exclusion will be kept and presented as part of the flow diagram.

The final search results will be outlined in a PRISMA flow diagram from the PRISMA-ScR statement, which will be accompanied by a narrative description of the process.

### Stage 4: Charting the data

This scoping review is designed to identify the range of the evidence available and represent this as a mapping of the identified data, without the act of synthesis or with particular reference to methodological quality of relevant studies ^24^. For data extraction the standardised template from the JBI methodology guidance for scoping reviews will be adapted for use ^19^.

Key information will be organised in categories based on data from organisational characteristics (evidence source details) e.g., name, country, role, and membership. Details extracted from the source of evidence; characteristics related to the methods (e.g. general description of the method of development), the clinical topics covered and approach to structure and presentation. Modes of dissemination will be recorded as well as implementation strategies.

These will be classified and categorised to generate a map of the methods used internationally to develop evidence-based guides for general practitioners and a narrative synthesis conducted.

As part of this process one reviewer will independently chart the data from the retrieved articles using the data charting form developed for this review ( Table 3). The second reviewer will check a sample of 20% of the charted data. They will then discuss the results and update the data charting form in an iterative process. Reasons for changes will be outlined and presented as an appendix as part of the review. If there are any inconsistencies these will be reviewed by a third reviewer.

**Table 3.** Data charting form.

**Scoping Review Details**
Scoping Review Title
Scoping Review Objectives
Scoping Review Questions
**Evidence Source Details and Characteristics**
Citation details (e.g. author/s, date, title, journal, volume, issue, pages)
Country
Organisation
**Details extracted from source of evidence**
Methods of Development
Topics covered
Structure/Presentation
Dissemination
Implementation

### Stage 5: Collating, summarising and reporting of results

Results will be reported using the PRISMA-ScR guidelines ^23^. Each research question will be reported separately and presented in a tabular form and as a narrative summary. This narrative description will be used to synthesise the study findings based on themes that are generated from the extracted data.

## Dissemination

We intend to disseminate the results through publication in a peer-reviewed journal and conference presentations.

## Study status

Database searches have been completed and title and abstract screening is currently underway.

## Ethics

Ethical approval is not required for this scoping review.

## Discussion

This scoping review will provide an overview of the evidence-based guidance produced and disseminated by GP professional organisations internationally. This scoping review can contribute to the evidence base for supporting GPs clinical decision making to benefit patient care. The findings of this scoping review will inform future research on the content, presentation dissemination and implementation of evidence-based guidance for GPs.

## Data availability

No data are associated with this article.
