# A scoping review protocol of evidence-based guidance and guidelines published by general practitioner professional organisations

**DOI:** 10.12688/hrbopenres.13268.3

**Published:** 2022-02-22

**Authors:** Emer O'Brien, Barbara Clyne, Susan M. Smith, Noirin O'Herlihy, Velma Harkins, Emma Wallace

**Affiliations:** 1 Department of General Practice, Royal College of Surgeons in Ireland, Dublin, D02H903, Ireland; 2 Irish College of General Practitioners, 4/5 Lincoln Place, Dublin 2, D02XR68, Ireland

**Keywords:** General practice, family practice, general practitioner, family practitioner, primary healthcare, practice guidelines, evidence based practice.

## Abstract

**Introduction: **General practitioners (GPs) strive to use a patient centered approach to achieve shared decision making by integrating clinical evidence, clinical judgement, and patient priorities. In order to achieve this standard of care, GPs require relevant, up to date and high quality evidence. Currently there is a gap in the literature regarding the role of GP professional organisations internationally in producing and publishing evidence based guidance and clinical guidelines for GPs. This protocol outlines a scoping review to identify what evidence-based guidance is produced by general practitioner professional organisations internationally in terms of topic content, the structure and methods used to develop guidance and ways of disseminating this guidance, to support general practice clinical decision making.

**Methods: **This scoping review will be conducted using the framework proposed by the Joanna Briggs Institute and the Preferred Reporting Items for Systematic Reviews and Meta-analysis extension for scoping reviews (PRISMA-ScR), will be used to guide the reporting. Two researchers will search electronic databases (Medline, Embase, Cochrane Library and Scopus), grey literature sources and contact international GP professional organisations directly to identify appropriate studies for inclusion. Key information will be categorised and classified to generate a summary of the methods used internationally to develop and implement evidence-based guides for general practitioners and a narrative synthesis will be conducted.

**Conclusions: **This scoping review will examine current practice internationally regarding the role of General Practice professional organisations in producing and publishing clinical guidelines and evidence based guidance to support general practitioner’s clinical decision making to benefit patient care.

## Introduction

General practitioners (GPs) require evidence-based guidance to support patient care ^1– 5^. GPs have a unique role in society, practicing medicine in the context of the family and community ^6^. GPs strive to use a patient centred approach to achieve shared decision making by integrating clinical evidence, clinical judgement, and patient priorities ^7, 8^.

There are differences internationally in healthcare systems and the cultural context in which GPs practice, but the role of the GP internationally has similarities in that GPs are ‘ *primarily responsible for the provision of comprehensive and continuing care to every individual seeking medical care irrespective of age, sex and illness ^9^* GPs in some countries have a gatekeeper role, authorising access to specialty care, hospital care, and some diagnostic tests ^10^.

Clinical practice guidelines are systematically developed statements, based on a thorough evaluation of the evidence, to assist practitioner and service users’ decisions about healthcare ^11^. However, a review of 45 UK guidelines reported that many guideline recommendations were based on studies with little or no relevance to primary care ^12^. GPs are more likely to use guidelines where the evidence is perceived to be applicable to primary care and where there has been GP input at the guideline development stage ^13^.

Internationally, GP professional organisations, as key GP opinion leaders, play a varied role in the clinical practice guideline ecosystem. First, in certain countries, GP professional organisations develop ‘de novo’ clinical guidelines. For example, the Dutch College of General Practice (NHG) develop *de novo* clinical guidelines for GPs across a range of primary care presentations and commonly managed conditions ^14^. Second GP professional organisations may be approached to endorse clinical practice guidelines developed by external organizations and groups ^15^. Third, GP professional organisations may disseminate materials to GPs based on national or international guidelines as part of an adopt or adapt approach ^16– 18^. While methodological guidance exists to support the process of adoption and adaption of guidelines, there is a need to have standardisation of this process to facilitate reproducibility and reduce duplication of effort.

Time pressure and increased workload are established barriers to GP implementation of clinical guidelines and evidence ^19^. GP professional organisations are well placed to support GPs to assimilate required evidence through the provision of easily accessible, high level clinical guideline summaries and evidence synopses. However what role, if any, GP organisations take in the dissemination of such evidence is unknown. This gap in the literature limits the ability of GP professional organisations to share both experience and expertise in how best to support GPs in their clinical decision making to support evidence-based patient care. 

 The aim of this scoping review is to identify what evidence-based guidance is published by GP professional organisations internationally to support GPs in their clinical decision making. The objectives are i) to identify the topics covered, both clinical and non-clinical; ii) to review the methods used to develop evidence-based guidance and/or clinical guidelines and how these guidance documents are structured and, iii) to explore how evidence is disseminated to GPs.

## Methods

### Scoping review framework

This scoping review will follow the framework proposed by the Joanna Briggs Institute (JBI) ^20– 23^. While, the overall conduct of the scoping review is informed by the JBI framework, the Preferred Reporting Items for Systematic Reviews and Meta-Analysis extension for scoping reviews (PRISMA-ScR) will be used to guide the reporting of the scoping review ^24^.

As this is a scoping review it will be designed to identify the range of the evidence available and will be represented as a mapping of the identified data, without the act of synthesis or particular reference to methodological quality of relevant studies ^25^.

### Stage 1: Identifying the research question

Overarching research question: What evidence-based guidance is published by general practice professional organisations to support GPs clinical decision making?

Objectives:

a. What are the content topics that the organisations are providing?

b. What are the methods for developing these guides?

c. What are the structures of the guides and how are they presented?

d. How are these guides disseminated to GPs?

### Stage 2: Identifying the relevant studies

Table 1 contains the eligibility criteria for the scoping review. Articles will be included where they are an evidence-based guidance document or guideline produced by a national general practice professional organisation. These guidance documents must support GPs clinical decision making and patient clinical care and be published in the last 10 years for currency. For the purposes of this scoping review ‘published’ refers to guidance documents that are made available by GP professional organisations, either freely on their websites or on a membership basis or through peer reviewed publications. These documents can be developed de novo or through adaptation processes. It does not include guidelines that are collaborations with or endorsed by other, non GP guideline producing organisations. No language restrictions will be applied.

**Table 1.** Eligibility criteria for inclusion in the review.

Inclusion Criteria	Rationale
• Evidence based guidance developed following a comprehensive review of the literature	Any evidence-based guidance or guideline produced by general practitioner (GP) professional organisations to support GP clinical decision-making. In order to be considered evidence-based, guidance documents will be included where they explicitly state they are based on a review of the literature (including systematic reviews, scoping reviews, rapid reviews, narrative reviews) Must be peer reviewed, reviewed by committee or experts. Definition of Evidence based guidelines *‘systematically developed statements to assist practitioner decisions about appropriate* *healthcare for specific clinical circumstances’* Evidence based as they *‘use the results of systematic literature reviews in formulating the* *recommendations’* ^26^
• Published by General Practice professional organisations	Publications must directly target GPs
• National	Regional programmes excluded
• Publications within the last 10 years	Most relevant
• No language restriction	
• For the purposes of this review the following definition of general practice is used	‘ *General practitioners/family doctors are specialist physicians trained in the principles of the discipline. They are personal doctors,* *primarily responsible for the provision of comprehensive and continuing care to every individual seeking medical care irrespective* *of age, sex and illness. They care for individuals in the context of their family, their community, and their culture, always respecting* *the autonomy of their patients. They recognise they will also have a professional responsibility to their community. In negotiating* *management plans with their patients they integrate physical, psychological, social, cultural and existential factors, utilising the* *knowledge and trust engendered by repeated contacts. General practitioners/family physicians exercise their professional role by* *promoting health, preventing disease and providing cure, care, or palliation. This is done either directly or through the services * *of others according to health needs and the resources available within the community they serve, assisting patients where* * necessary in accessing these services. They must take the responsibility for developing and maintaining their skills, personal* * balance and values as a basis for effective and safe patient care’* ^9^.
• Patient clinical care	Exclude guidance relating to practice management and other non- clinical topics

## Search strategy

Given the nature of the topic, the fact that many guides may not be published as peer reviewed publications and considerable heterogeneity of the nomenclature (e.g. guides versus guidance versus clinical guidelines), the search strategy will identify both peer reviewed studies and grey literature. This will be achieved through a combination of bibliographic database searching, grey literature searching and contacting key informants. All these processes will be conducted in tandem and are not sequential.

### Bibliographic database searching

The bibliographic database searching will follow a three step strategy, as per JBI ^20^. A copy of the search strategy is shown in Table 2.

**Table 2.** Search strategy.

	Ovid MEDLINE(R) and Epub Ahead of Print, In-Process, In-Data-Review & Other Non-Indexed Citations, Daily and Versions(R) 1946 to March 09, 2021	
1	general practice.mp. or exp Family Practice/ or exp General Practice/	97226
2	(general ADJ1 practitioner) OR (general ADJ1 practitioners) OR (family ADJ1 practi?e) OR (family ADJ1 physician*)	126886
3	primary health care.mp. or exp Primary Health Care/	310657
4	1 OR 2 OR 3	
5	exp Practice Guideline/ OR exp Practice Guidelines as Topic/ OR (practice ADJ2 guideline$)	166143
6	((quick adj2 reference adj2 guide*) or (quick adj2 reference) or (evidence adj1 reference) or (evidence adj1 guide*) or ("evidence based" adj1 reference) or ("evidence based" adj1 guide)).mp.	1520
7	5 OR 6	167271
8	4 AND 7	12186
8	LIMIT 8 to 2010–2021	6175

The **first step**, the limited search, will include searching two appropriate online databases ( Medline and Embase). An analysis of the text words in the titles and abstracts of retrieved papers will be conducted, and of the index terms used to describe the articles.

The **second step** will use all identified key words and index terms to perform a second search of all the following databases: Medline, Embase, Cochrane Library and Scopus to identify peer reviewed research papers relating to our aim. This step will be conducted with input from an information specialist.

**Thirdly,** reference lists of included articles will be searched for additional relevant articles.

### Grey literature search

Grey literature searching will be conducted by searching ‘Guideline Central’ and ‘NHS Evidence Search’ using combinations of the key words and index terms used in the bibliographic database search.

### Contacting key informants

Key informants in GP professional organisations, as per the definition of general practice outlined in Table 1, will be contacted through professional links via the Irish College of General Practitioners (ICGP) and links with the European Society for Quality and Safety in Practice (EQuiP) in WONCA Europe.

The final included studies for screening will be downloaded to a reference management software package ( EndNote X9) and duplicates removed.

### Stage 3: Study selection

Titles and abstracts will be screened for inclusion against the inclusion criteria for the review ( Table 1). For those that appear to meet the inclusion criteria, full text articles will be retrieved and screened against the inclusion criteria. Those articles that fulfil all the inclusion criteria will be included in the review.

The above steps will be completed by two reviewers (EOB and SD). They will work independently initially and then come together to compare results. Any discrepancies will be resolved by consensus and if consensus is not reached will be referred to a third reviewer (EW).

Studies that do not meet the inclusion criteria will be excluded. Reasons for the exclusion will be kept and presented as part of the flow diagram.

The final search results will be outlined in a PRISMA flow diagram from the PRISMA-ScR statement, which will be accompanied by a narrative description of the process.

### Stage 4: Charting the data

This scoping review is designed to identify the range of the evidence available and represent this as a mapping of the identified data, without the act of synthesis or with particular reference to methodological quality of relevant studies ^25^. For data extraction the standardised template from the JBI methodology guidance for scoping reviews will be adapted for use ^20^.

Key information will be organised in categories based on data from organisational characteristics (evidence source details) e.g., name, country, role, and membership. Details extracted from the source of evidence; characteristics related to the methods (e.g. general description of the method of development), the clinical topics covered and approach to structure and presentation. Modes of dissemination will be recorded as well as implementation strategies.

These will be classified and categorised to generate a map of the methods used internationally to develop evidence-based guides for general practitioners and a narrative synthesis conducted.

As part of this process one reviewer will independently chart the data from the retrieved articles using the data charting form developed for this review ( Table 3). The second reviewer will check a sample of 20% of the charted data. They will then discuss the results and update the data charting form in an iterative process. Reasons for changes will be outlined and presented as an appendix as part of the review. If there are any inconsistencies these will be reviewed by a third reviewer.

**Table 3.** Data charting form.

Scoping Review Details
Scoping Review Title
Scoping Review Objectives
Scoping Review Questions
Evidence Source Details and Characteristics
Citation details (e.g. author/s, date, title, journal, volume, issue, pages)
Country
Organisation
Guideline type
Details extracted from source of evidence
Methods of Development
Update (frequency and method)
Topic covered
Structure/Presentation
Dissemination
Implementation

### Stage 5: Collating, summarising and reporting of results

Results will be reported using the PRISMA-ScR guidelines ^24^. Each research question will be reported separately and presented in a tabular form and as a narrative summary. This narrative description will be used to synthesise the study findings based on themes that are generated from the extracted data.

## Dissemination

We intend to disseminate the results through publication in a peer-reviewed journal and conference presentations.

## Study status

Database searches have been completed and title and abstract screening is currently underway.

## Ethics

Ethical approval is not required for this scoping review.

## Discussion

This scoping review will provide an overview of the evidence-based guidance produced and disseminated by GP professional organisations internationally. This scoping review can contribute to the evidence base for supporting GPs clinical decision making to benefit patient care. Furthermore, the findings of this research can identify potential areas for standardisation internationally to facilitate reproducibility and reduce duplication of effort. The findings of this scoping review will inform future research on the content, presentation dissemination and implementation of evidence-based guidance for GPs.

## Data availability

No data are associated with this article.
