# Supplementary material for: A scoping review protocol of evidence-based guidance and guidelines published by general practitioner professional organisations
Source: HRB Open Res. 2022 Feb 22;4:53. Originally published 2021 May 18. [Version 3] doi: 10.12688/hrbopenres.13268.3 (PMC8866908; doi:10.12688/hrbopenres.13268.3)
Supplement: Peer review history

# Peer review: Reviewer response for version 3

**DOI:** 10.21956/hrbopenres.14738.r31528

**Published:** 2022-02-23

**Authors:** Jako Burgers

**Affiliations:** 1 Dutch College of General Practitioners, Utrecht, The Netherlands; 2 Care and Public Health Research Institute, Maastricht University, Maastricht, The Netherlands

I am happy with this new version.

Is the study design appropriate for the research question?

Yes

Is the rationale for, and objectives of, the study clearly described?

Yes

Are sufficient details of the methods provided to allow replication by others?

Yes

Are the datasets clearly presented in a useable and accessible format?

Yes

Reviewer Expertise:

Clinical practice guidelines, general practice, primary care, person-centered care

I confirm that I have read this submission and believe that I have an appropriate level of expertise to confirm that it is of an acceptable scientific standard.

# Peer review: Reviewer response for version 2

**DOI:** 10.21956/hrbopenres.14668.r31352

**Published:** 2022-02-01

**Authors:** Jako Burgers

**Affiliations:** 1 Dutch College of General Practitioners, Utrecht, The Netherlands; 2 Care and Public Health Research Institute, Maastricht University, Maastricht, The Netherlands

This is an interesting study identifying clinical practice guidelines targeting general practice and the role of its professional organisations worldwide. I have one major and a few minor comments.

 One major comment concerns the search strategy. The authors start with searching online databases and then targeting professional organisation contacts. I would suggest reversing the order. As general practice guidelines are often not included in medical databases, the best way to identify them is to contact professional organisations, for instance, by using the WONCA Network and/or GIN Network. The websites of member organisations could be explored for guidelines published or links to guideline databases. In addition but as a second step, online databases could be searched. In contrast to systematic reviews, a scoping review does not aim to be complete, so efforts could be saved by limiting the search strategy. If the authors still decide to start with searching the online databases, it would be recommended to only follow step 1 as exploration. 

 Minor comments are:

1. There is no definition provided of 'guidance'. NICE uses this as an umbrella term, including (evidence-based) guidelines, others use the term to describe all other tools to support decision making in clinical practice that are not evidence based, in contrast to guidelines. I would suggest limiting the review to guidelines, which are evidence-based by definition (according to IOM).
2. The term 'published' also needs clarification. This can be on paper or digital. In the legal context, the term 'endorsed' or 'approved' is more relevant than 'published' as this means that the professional needs to know and to follow the guideline in principle. The term 'published' is more vague. It would be interesting to focus on those guidelines that are part of a programme or set of guidelines used for education, training, and evaluation. As this is a scoping review, the scope can be changed or refined during the study if more focus is desirable to answer the research question.
3. The search strategy identifying general practice could be expanded with terms as 'Primary Health Care' or 'Physicians, Primary Care'. This is broader than 'General Practice' and 'Family Practice' but some countries do not use these terms. 
4. In Discussing the potential impact of the findings of this study, the authors could discuss the opportunities to share guidelines and their supporting evidence internationally to reduce duplication of efforts. 

Is the study design appropriate for the research question?

Yes

Is the rationale for, and objectives of, the study clearly described?

Yes

Are sufficient details of the methods provided to allow replication by others?

Yes

Are the datasets clearly presented in a useable and accessible format?

Yes

Reviewer Expertise:

Clinical practice guidelines, general practice, primary care, person-centered care

I confirm that I have read this submission and believe that I have an appropriate level of expertise to confirm that it is of an acceptable scientific standard, however I have significant reservations, as outlined above.

# Peer review: Reviewer response for version 2

**DOI:** 10.21956/hrbopenres.14668.r30837

**Published:** 2021-11-11

**Authors:** Tony Foley

**Affiliations:** 1 Department of General Practice, University College Cork, Cork, Ireland

Many thanks for reviewing my comments and making minor revisions.

 I have no further comments to make.

Is the study design appropriate for the research question?

Yes

Is the rationale for, and objectives of, the study clearly described?

Yes

Are sufficient details of the methods provided to allow replication by others?

Yes

Are the datasets clearly presented in a useable and accessible format?

Yes

Reviewer Expertise:

general practice, medical education, dementia care, scoping review, qualitative research

I confirm that I have read this submission and believe that I have an appropriate level of expertise to confirm that it is of an acceptable scientific standard.

# Peer review: Reviewer response for version 1

**DOI:** 10.21956/hrbopenres.14441.r30449

**Published:** 2021-10-18

**Authors:** Katherine Checkland

**Affiliations:** 1 Division of Population Health, Health Services Research and Primary Care, University of Manchester, Manchester, UK

I have some concerns about this study, and its potential to provide useful evidence. In particular, I do not feel that the rationale for focusing upon the role of GP professional organisations in guideline production is well described. I think that the researchers are suggesting that guidelines produced specifically by GP professional organisations will somehow be better/more applicable than guidelines produced by other organisations, but this case is not made. In addition, I think that current professional focus is generally more upon how you manage patients with more than one condition, and in these circumstances, disease specific guidelines are less applicable. Finally, the authors do not provide any research questions. I would recommend that they do this before embarking on their review

Is the study design appropriate for the research question?

No

Is the rationale for, and objectives of, the study clearly described?

Partly

Are sufficient details of the methods provided to allow replication by others?

Yes

Are the datasets clearly presented in a useable and accessible format?

Not applicable

Reviewer Expertise:

I am a GP and qualitative researcher

I confirm that I have read this submission and believe that I have an appropriate level of expertise to state that I do not consider it to be of an acceptable scientific standard, for reasons outlined above.

# Response

**Published:** 2021-11-02

**Authors:** Emer O'Brien

**Affiliations:** Department of General Practice, RCSI, Dublin, Ireland

Many thanks for agreeing to review this Scoping Review protocol. We address your comments on a point by point basis below.

***Comment***

 I have some concerns about this study, and its potential to provide useful evidence. In particular, I do not feel that the rationale for focusing upon the role of GP professional organisations in guideline production is well described. I think that the researchers are suggesting that guidelines produced specifically by GP professional organisations will somehow be better/more applicable than guidelines produced by other organisations, but this case is not made.

***Response***

 The rationale for focusing on GP professional organisations is to examine current practice internationally regarding the role of GP organisations in producing and publishing clinical guidelines and evidence based guidance for GPs. There is currently a lack of evidence as to what is being published by GP professional organisations internationally in terms of evidence based guidance and clinical guidelines for GPs to use in their clinical practice. Our initial literature search shows international variation in the role of GP professional organisations in publishing guidelines and evidence based guidance for GPs. For instance in the Netherlands, de novo clinical guidelines are developed and published by the Dutch College of GPs on a range of relevant topics whereas in other countries such as the UK clinical guidelines, aimed at GPs are published by central clinical guideline agencies such as NICE and SIGN while the Royal College of GPs focuses on the publication of other evidence based guidance for GPs.

*Internationally, GP professional organisations, as key GP opinion leaders, play a varied role in the clinical practice guideline ecosystem. First, in certain countries, GP professional organisations develop ‘de novo’ clinical guidelines. Second GP professional organisations may be* *approached to endorse clinical practice guidelines developed by external organizations and groups.* *Third, GP professional organisations may disseminate materials to GPs based on national or international guidelines as part of an adopt or adapt approach. While methodological guidance exists to support the process of adoption and adaption of guidelines, there is a need to have standardisation of this process to facilitate reproducibility and reduce duplication of effort.*

 Evidence shows that involving clinicians in guideline production relevant to their clinical practice and understanding the context in which the guidelines are to be implemented are very important aspects of ensuring clinical guidelines are implemented in practice. Understanding the role of GP professional organisations internationally in the production and publishing of clinical guidelines can inform best practice in this area and lessons can be shared regarding how different countries approach this issue and how that impacts on implementation.

*GPs are more likely to use guidelines where the evidence is perceived to be applicable to primary care and where there has been GP input at the guideline development stage*

 To capture the implementation of guidelines published by GP professional organisations internationally a key informant survey of GP professional organisations internationally will be conducted to further inform this scoping review. This will focus on the methods employed by different GP professional organisations in developing guidelines/evidence based guidance, how this is disseminated to GPs and the processes supporting implementation in clinical practice. This approach will give a rounded view of the utility of different approaches to guideline production and publication by GP professional organisations internationally.

 The introduction has been updated to include a discussion of the barriers to guideline implementation and the possible role GP professional organisation have in facilitating their use.

*Time pressure and increased workload are established barriers to GP implementation of clinical guidelines and evidence.*

*GP professional organisations are well placed to support GPs to assimilate required evidence through the provision of easily accessible, high level clinical guideline summaries and evidence synopses. However what role, if any, GP organisations take in the dissemination of such evidence is unknown. This gap in the literature limits the ability of GP professional organisations to share both experience and expertise in how best to support GPs in their clinical decision making to support evidence-based patient care.*

***Comment***

 In addition, I think that current professional focus is generally more upon how you manage patients with more than one condition, and in these circumstances, disease specific guidelines are less applicable.

***Response***

 Multimorbidity is one important area for GPs to have access to up to date information and there are already a number of guidelines developed to support GP decision making in this area e.g. UK NICE guideline for the clinical assessment and management of multimorbidity.

 This scoping review will provide an overview of the topics that GP professional organisations develop clinical guidelines and evidence based guidance for GPs and will answer the question as to what topics GP professional organisations are prioritising for their GP members.

***Comment***

 Finally, the authors do not provide any research questions. I would recommend that they do this before embarking on their review

***Response***

 The overarching research question and objectives for this scoping review are outlined in the Methods section; scoping review framework; stage 1; identifying the research question.

 For ease of reference we include the research question and review objectives again here;

*Overarching research question:* *What evidence-based guidance is published by general practice professional organisations to support GPs clinical decision making?*

*Objectives:*

*a. What are the content topics that the organisations are providing?*

*b. What are the methods for developing these guides?*

*c. What are the structures of the guides and how are they presented?*

*d. How are these guides disseminated to GPs?*

***Comment***

**Is the rationale for, and objectives of, the study clearly described?**

 Partly

***Response***

 Given the updates outlined above we would hope that the rationale for the study is now more clearly described.

***Comment***

**Is the study design appropriate for the research question?**

 No

***Response***

 Given the amendments made to more clearly outline the overall rationale for this study we hope that the reasoning for the choice of study design is also clearer now. A scoping review is defined as a review that is designed to address a broad research question by mapping a body of literature in that area and also to identify gaps in the research knowledge area (Peters, Godfrey et al. 2015) Scoping reviews are used not only to determine the extent of the research in a particular area but also the way the research was conducted. As such, we believe it is the appropriate design to answer the research question for this review; ‘What evidence based guidance and guidelines is published by general practice professional organisations to support GPs clinical decision making’. By supplementing the scoping review of the literature with a targeted survey of GP professional organisations internationally the review will be able to provide data on other key aspects of guideline production, dissemination and implementation by GP professional organisations.

# Peer review: Reviewer response for version 1

**DOI:** 10.21956/hrbopenres.14441.r29630

**Published:** 2021-06-10

**Authors:** Tony Foley

**Affiliations:** 1 Department of General Practice, University College Cork, Cork, Ireland

Thanks for the opportunity to review this interesting scoping review protocol.

**Abstract:** Consider adding a line to briefly explain the importance of this topic - evidence-based guidelines. The aim given here is to 'identify what evidence-based guidance is produced...'. Consider expanding on this identify studies/content topics/methods of development/dissemination.

**Introduction:**

 While the importance of evidence-based guidance to support patient care is made and also the different approaches used internationally, I feel the authors need to expose and highlight the gap in research. Have any similar reviews been undertaken before or are any scoping/systematic reviews currently ongoing? What literature is out there on the aims/objectives of this study?

 'The role of the GP in the healthcare system also has an impact'.....Consider expanding on this sentence to explain to the reader what impact is being discussed.

 '​​​​​​GPs who are gatekeepers are more likely to require GP orientated guidelines'...Perhaps this statement could also be further explained?

**Methods:**

 Search Strategy: Table 1 Eligibility criteria - consider adding rationale why you're excluding papers with <=2 authors. Is that necessary? 

 Contacting GP professional organisations - Can you further identify which countries/regions will be included in this contact and justify why?

 Has the protocol been registered? e.g. open science framework/prospero.

Is the study design appropriate for the research question?

Yes

Is the rationale for, and objectives of, the study clearly described?

Yes

Are sufficient details of the methods provided to allow replication by others?

Yes

Are the datasets clearly presented in a useable and accessible format?

Yes

Reviewer Expertise:

general practice, medical education, dementia care, scoping review, qualitative research

I confirm that I have read this submission and believe that I have an appropriate level of expertise to confirm that it is of an acceptable scientific standard.

# Response

**Published:** 2021-11-02

**Authors:** Emer O'Brien

**Affiliations:** Department of General Practice, RCSI, Dublin, Ireland

Thanks for the opportunity to review this interesting scoping review protocol.

*Response*

 Many thanks for agreeing to review this Scoping Review protocol.

**Abstract:** Consider adding a line to briefly explain the importance of this topic - evidence-based guidelines. The aim given here is to 'identify what evidence-based guidance is produced...'. Consider expanding on this identify studies/content topics/methods of development/dissemination.

*Response*

 Abstract has been amended to include suggestions above.

 See paragraph 1 Introduction and paragraph 3 conclusion of Abstract

*‘in terms of topic content,* *the structure and methods used to develop* *guidance* *and ways of disseminating this guidance’*

**Introduction:**

 While the importance of evidence-based guidance to support patient care is made and also the different approaches used internationally, I feel the authors need to expose and highlight the gap in research. Have any similar reviews been undertaken before or are any scoping/systematic reviews currently ongoing? What literature is out there on the aims/objectives of this study?

*Response*

 There is currently a lack of evidence as to what is being published by GP professional organisations internationally in terms of evidence based guidance and clinical guidelines. There appears to be international variation in the role of GP professional organisations in publishing guidelines and evidence based guidance. For instance in the Netherlands, de novo clinical guidelines are developed and published by the Dutch College of GPs on a range of relevant topics whereas in other countries such as the UK, clinical guidelines aimed at GPs are published by central clinical guideline agencies such as NICE and SIGN while the Royal College of GPs focuses on the publication of other evidence based resources for GPs.

 See paragraph 3 and 4 of Introduction, research gap highlighted by expanding on the above points and also making clear the rationale for this review.

*Clinical practice guidelines are systematically developed statements, based on a thorough evaluation of the evidence, to assist practitioner and service users’ decisions about healthcare.*

*However, a review of 45 UK guidelines reported that many guideline recommendations were based on studies with little or no relevance to primary care.*

*GPs are more likely to use guidelines where the evidence is perceived to be applicable to primary care and where there has been GP input at the guideline development stage.*

 'The role of the GP in the healthcare system also has an impact'.....Consider expanding on this sentence to explain to the reader what impact is being discussed.

*Response*

 Introduction paragraph 2 has been updated to put both healthcare system and role of GP as gatekeeper into context

*‘There are differences internationally in healthcare systems and the cultural context in which GPs practice, but the role of the GP internationally has similarities in that GPs are ‘primarily responsible for the provision of comprehensive and continuing care to every individual seeking medical care irrespective of age, sex and illness’*

 '​​​​​​GPs who are gatekeepers are more likely to require GP orientated guidelines'...Perhaps this statement could also be further explained?

*Response*

 See above

**Methods:**

 Search Strategy: Table 1 Eligibility criteria - consider adding rationale why you're excluding papers with <=2 authors. Is that necessary? 

*Response*

 Thank you. The author number restriction has been removed.

 Contacting GP professional organisations - Can you further identify which countries/regions will be included in this contact and justify why?

*Response*

 The Key Informant Survey will be circulated to GP professional organisations colleges in Europe, USA, Canada, Australia and New Zealand. We are targeting national GP professional organisations in countries that have a GP system in line with the WONCA definition of General/Family Practice (Table 1 of protocol).

 See Methods, Search Strategy Stage 2 for updated text.

*Various avenues will be utilised to contact key informants including WONCA Europe contacts.*

 Has the protocol been registered? e.g. open science framework/prospero.

*Response*

 Prospero does not accept registration of Scoping Review Protocols.

 The review has been registered with Open Science Framework.
